# Letter to the editor: esophageal inflammatory fibroid polyp in a Nigerian

**DOI:** 10.4314/ahs.v22i2.39

**Published:** 2022-06

**Authors:** Aderemi Oluyemi, Emmanuel Oguntebi, Nicholas Awolola

**Affiliations:** 1 ReMay Consultancy & Medical Services, Ikeja, Lagos State, Nigeria; 2 Pathology Department, Clinix HealthCare, Ilupeju, Lagos State, Nigeria; 3 College of Medicine, University of Lagos, Lagos State, Nigeria

Dear Editor,

In 2016, we published a case report in your esteemed journal detailing the rare occurrence of a rectal inflammatory fibroid polyp (IFP)^1^. We had noted then that certain sites in the gastrointestinal (GI) tract were not usual for this benign tumour to occur. This letter is written to document yet another such rare IFP lesion in an even rarer still region- the oesophagus.

**Figure 1:**
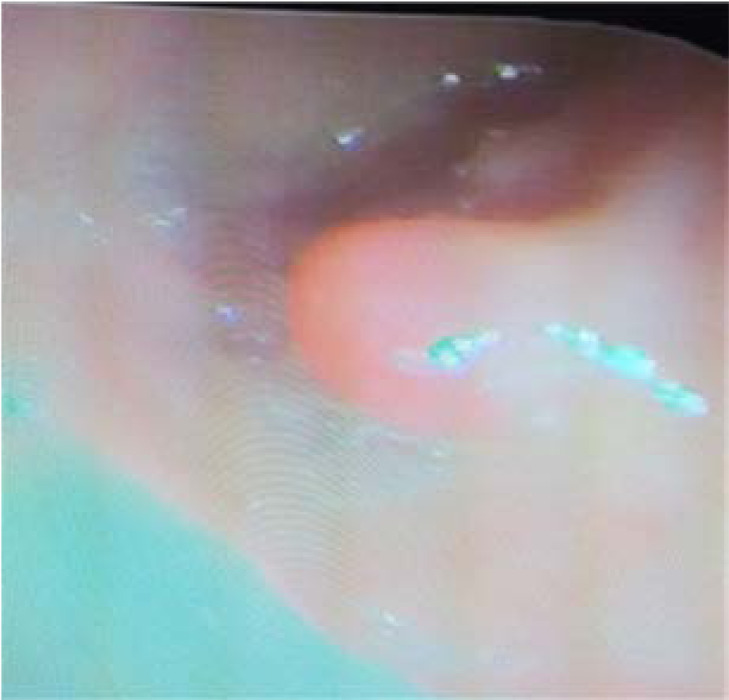
Endoscopic image of the oesophageal polyp.

The patient is a 76 year old, retired teacher who presented for gastroscopy on account of recent onset pain in the upper abdomen and mild anaemia. Two weeks prior to the onset of his pain, he had recently been placed on some non-steroidal anti-inflammatory drugs in additional to the daily Aspirin (at 75 mg) that had been prescribed a few years back. He had no history of smoking. A tiny, sub-mucosal growth was noted in the throat without any overlying mucosa abnormalities (Figure 1).

**Figure 2:**
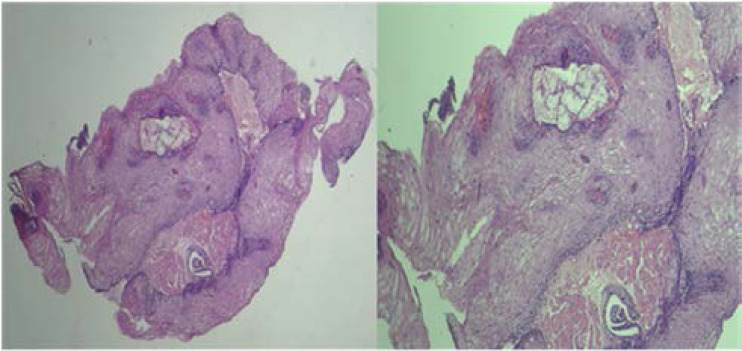
Image showing hyperplastic overlying stratified squamous epithelium, island of mucous oesophageal glands and underlying myofibroblasticstroma. (Haematoxylin and Eosin stain; Original magnification- Image on the Left X 40 & Image on the Right X 400).

The stomach featured antral erosions and gastritis. He was then asked about any oesophageal symptoms but responded that nothing of note bothered in the throat apart from intermittent feeling of lump there. The oesophageal lesion was excised and the histopathological report came back as IFP (Figure 2).

The computed tomography (CT) done at the same centre detected the lesion and noted the absence of infiltration or any other feature of malignancy. A call was put through to the referring physician after the results of the pathology were received. The doctor confirms that the patient firmly refused any suggestion of endoscopic ultrasonography or other intervention of any kind. The repeat CT scan done 3 years after the initial scope did not show any appreciable change.

This represents yet another unique finding. IFPs may occur in this region but that is a rarity indeed. In previous descriptions, dysphagia was the predominant complaint^2^,^3^. But more alarming presentations like massive haemorrhage^4^ and obstruction of the airways^5^ have been described. The response to surgical and or endoscopic intervention is splendid. This is comforting as the tumour itself is benign with little or no known malignant potential.
